# Estimating front-wave velocity of infectious diseases: a simple, efficient method applied to bluetongue

**DOI:** 10.1186/1297-9716-42-60

**Published:** 2011-04-20

**Authors:** Maryline Pioz, Hélène Guis, Didier Calavas, Benoît Durand, David Abrial, Christian Ducrot

**Affiliations:** 1 Institut National de la Recherche Agronomique, Centre de Clermont-Ferrand Theix, Unité d'Epidémiologie Animale, St Genès Champanelle, France; 2 CIRAD, UMR Contrôle des maladies, F-34398 Montpellier, France; 3 French Agency for Food, Environnemental and Occupational Health Safety, Lyon, France; 4 French Agency for Food, Environnemental and Occupational Health Safety, Maisons-Alfort, France

## Abstract

Understanding the spatial dynamics of an infectious disease is critical when attempting to predict where and how fast the disease will spread. We illustrate an approach using a trend-surface analysis (TSA) model combined with a spatial error simultaneous autoregressive model (SAR_err _model) to estimate the speed of diffusion of bluetongue (BT), an infectious disease of ruminants caused by bluetongue virus (BTV) and transmitted by *Culicoides*. In a first step to gain further insight into the spatial transmission characteristics of BTV serotype 8, we used 2007-2008 clinical case reports in France and TSA modelling to identify the major directions and speed of disease diffusion. We accounted for spatial autocorrelation by combining TSA with a SAR_err _model, which led to a trend SAR_err _model. Overall, BT spread from north-eastern to south-western France. The average trend SAR_err_-estimated velocity across the country was 5.6 km/day. However, velocities differed between areas and time periods, varying between 2.1 and 9.3 km/day. For more than 83% of the contaminated municipalities, the trend SAR_err_-estimated velocity was less than 7 km/day. Our study was a first step in describing the diffusion process for BT in France. To our knowledge, it is the first to show that BT spread in France was primarily local and consistent with the active flight of *Culicoides *and local movements of farm animals. Models such as the trend SAR_err _models are powerful tools to provide information on direction and speed of disease diffusion when the only data available are date and location of cases.

## Introduction

Understanding the spatial dynamics of an infectious disease is critical when attempting to predict where and how fast the disease will spread. The use of simulation modelling for estimating the spread of infectious animal diseases has now become common [1-4]. However, most of these modelling approaches are complex, based on spatially explicit, stochastic, state-transition or network models [2,5-7] or on diffusion equations [8,9]. While such modelling approaches require precise knowledge on the model parameters, available data on emerging animal diseases are often restricted to case reports providing only date and location. These limited data nevertheless can provide useful information on the direction and speed of disease diffusion and be used to estimate front-wave velocity of an infectious disease. We illustrate an approach using a trend-surface analysis (TSA) model combined with a simultaneous autoregressive spatial error (SAR_err_) model to estimate the speed of diffusion of bluetongue (BT).

BT is a non-contagious, infectious, viral disease of ruminants caused by bluetongue virus (BTV) and transmitted by biting midges (*Culicoides*) [10]. 24 serotypes of BTV have now been described. Until recently, BT was thought to be restricted to tropical regions and southern Europe where competent *Culicoides *species vectors are present [11]. The large-scale, BTV serotype 8 (BTV-8) epidemic in north-western Europe in 2006-2008 consequently surprised the veterinary community and caused major economic losses [12]. Contrary to what was previously thought, the abundant local *Culicoides *species in north-western Europe are vector competent [13,14]. Disease progression was thus rapid: 2 000 infected farms across Belgium, Germany, The Netherlands, France, and Luxembourg in 2006 [12], more than 30 000 farms in 2007 with an expansion in range to Denmark, the United Kingdom, Switzerland, and the Czech Republic [12,15].

While active flight of infected *Culicoides *is responsible for local propagation of BT, the movement of viremic animals and passive flight of infected *Culicoides *carried by the wind are responsible for long distance (>100 km) dissemination of the infection [16]. Quantification of the respective importance of local spread and long distance dissemination of BT currently is lacking. Gerbier et al. [17] suggested that the effect of wind was probably negligible for BT diffusion in 2006 and that local spread of BTV-8 can be explained mainly by active flight of *Culicoides*. Moreover, Mintiens et al. [18] showed that control measures implemented on animal movements failed to stop further spread of BTV because it is impossible to limit vector movements. The authors, however, noted that an absence of control measures probably would have resulted in an even wider and faster spread.

In a first step to gain further insight into the spatial transmission characteristics of BTV-8, we used 2007-2008 clinical case reports in France and TSA combined to spatial error modelling to identify the major directions and speed of diffusion of the BT epidemic.

## Materials and methods

### Compilation of BT clinical cases

We used BT cases recorded by the Direction Générale de l'Alimentation of the French Ministry of Agriculture, Food and Fishing in 2007-2008 to assess front-wave velocity. A case was defined as a bovine herd or an ovine or goat flock in which BT was clinically suspected and later confirmed by serological or virological analyses. Our analysis was performed on a municipality basis (the smallest administrative unit in France). Cases with missing date of record or location data were discarded, leaving 33 042 cases in 12 620 municipalities belonging to 82 departments (French administrative unit of a median surface area of 5 985 km^2^). In 6.9% (*n *= 2 279) of these cases, the date of report was missing, however, the date of serological or virological confirmation was available. We were able to include these cases by extrapolating the date of report from the date of serological or virological confirmation. We did so by considering the 30 263 clinical cases with both date of report and date of confirmation, and verifying that the date of report and date of serological or virological confirmation were strongly correlated (*r^2 ^*= 0.99) with a mean delay of 6 days (0-152), and 95% of the delay being lower than 19 days.

**Figure 1:**
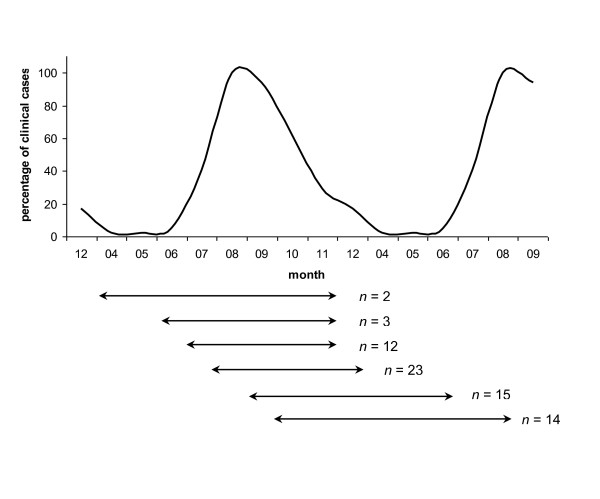
**Period of time during which we included municipalities that reported their first clinical BTV-8 case**. The curve represents the distribution of the clinical cases reported per month over 2007-2008, expressed in percentage compared to the month with the highest number of cases, i.e., August. The percentage of clinical cases represented on the vertical axis was thus 100% in August. The horizontal lines under the graph symbolize the time period during which we included the municipalities that reported their first clinical case of BT, in relation to the month of report of the first clinical case in the department. All observed situations are plotted. *n *represents the number of departments. For example, the first horizontal line means that for the two departments that reported their first clinical case in April, we included in our dataset the departments' municipalities that reported their first clinical case from April to November (included).

The data from the 33 042 clinical cases were then reduced to the first report of a case for each municipality. In the northern and central part of France, the French entomological surveillance reported only 7, 2 and 31 *Culicoides *captured in the 39, 34 and 46 traps set in January, February and March 2008, respectively (T. Balenghien, unpublished). Based on these entomological data and literature [13,19], we considered that *Culicoides *activity was negligible from January to March, and thus excluded these three months from the subsequent analyses. As clinical signs of BT can be missed [16,20], the disease may pass unnoticed in an infected herd over several weeks. To limit the bias due to unnoticed cases, we discarded municipalities with a very late first clinical case report in relation to the first clinical case report in the department. To do so, we estimated the mean length of a French department to be 105 km. Based on a mean velocity of 10 km per week [17], BT would pass through a French department within 75 days under optimal conditions. We added 75 more days to allow infected *Culicoides *to reach less accessible municipalities within the department. One hundred fifty days would thus be the restrictive delay to consider. However, as BT transmission depends on *Culicoides *density and activity, we took into account the month of the first case report of the department to balance the period of time during which we included municipalities depending on the month of the first case report in the department. The period of time during which we included municipalities with their first cases in relation to the month of the first case report in their department are presented Figure 1. The curve represents the distribution of the clinical cases reported per month over 2007-2008. The horizontal lines show the period of time during which we included the municipalities in each department depending on the month of the first clinical case report in the department. When the first clinical case report of the department occurred during the less favourable periods for vector activity (i.e., in April and October), we included municipalities for a longer period of time than when it occurred during the months of the peak of *Culicoides *activity (i.e., in July and August). In all, 1 627 municipalities in 51 departments were considered as having a late first case report and were discarded.

**Figure 2:**
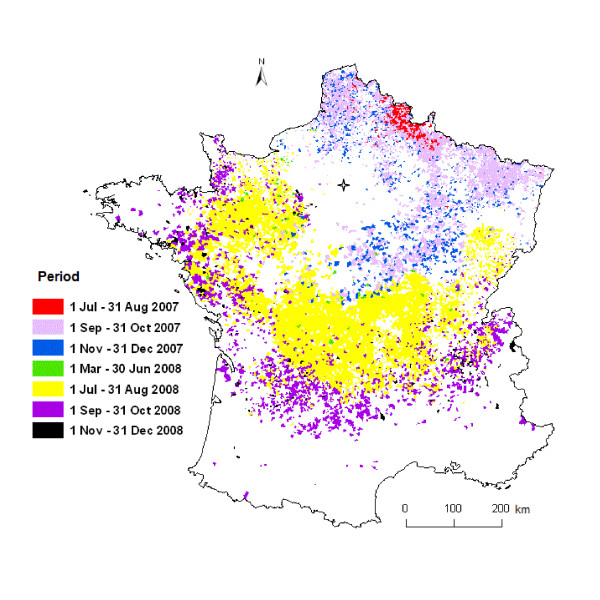
**French municipalities with at least one clinical case of BT reported in 2007-2008 (*n *= 10 994)**. Blank areas are areas where no or incomplete clinical cases were reported. Paris is represented by a star. Four periods were defined based on the ecology of *Culicoides *and BTV transmission rate: 1 July - 31 August, 1 September - 31 October, 1 November - 31 December, and 1 March - 30 June.

Our study consequently was based on 10 994 municipalities in 82 French departments which reported at least one case of BT (Figure 2).

### Trend-surface analysis

TSA is a least squares regression method used to study diffusion processes in space and time [21]. A surface pattern can be constructed by mapping the specific timing of events at each (X, Y) coordinate in two dimensions. The general procedure is described in Unwin [22]. Briefly, the method uses a model with power series polynomials, fitting linear, quadratic, cubic, and higher order trend-surfaces to the data [21]. The shape and flexibility of the trend surface are determined by the order of the polynomial chosen as the model [23]. A first-order polynomial restricts the trends to a plane through the data. Second-order polynomial models allow for curvature over the entire data set, while higher-order models allow for much more local curvature in the fitted surface [24]. In health research, TSA is a simple method which aims to capture the generalized direction(s) and speed(s) of the propagating wave of an infectious disease [24]. It previously has been used to assess the front-wave velocity of rabies and plague and to identify the pattern of disease diffusion [8,23-25].

In our study, a polynomial surface was fitted to the set of spatially distributed times of first BT clinical case detection across the 10 994 municipalities. The (X, Y) coordinates of the municipality centroids were uploaded into ArcGIS software v.9.1 (ESRI Inc.). Original geographical coordinates were expressed in meters, *Lambert II étendu*. They were then translated into (X, Y) coordinates with the origin adjusted to the area of BT introduction. This area, referred to as the Area of First Infection (AFI), was identified in Gerbier et al. [17] as the border area between The Netherlands, Germany, and Belgium (latitude = 2 669 013 m; longitude = 3 726 278 m). Retrospective preliminary reports on the first BTV-8 outbreaks in Belgium and Germany indicated that the first clinical signs appeared between 17 July and 5 August 2006 [16,26]. We therefore considered 17 July 2006 as the date of BT introduction in the AFI.

A model of the form t = β_0 _+ β_1_X + β_2_Y + β_3_X^2 ^+ β_4_XY + β_5_Y^2 ^+ ε was used to estimate linear and quadratic surfaces by least squares. In this model, t is the number of days to the BT introduction in 2006, β_i _are the fitted parameters, X and Y are the geographic coordinates of the municipality centroids adjusted to the area of BT introduction in Europe, and ε represents the error term. The main caveat for significance testing in TSA is that spatial autocorrelation in the residuals is almost always present to a certain extent by the nature of the spatial data [22]. In our study, residuals of the TSA models showed autocorrelation (Moran's I statistic = 0.3894, *p *< 0.001). Among the numerous methods available to deal with spatial autocorrelation, autoregressive models incorporate spatial autocorrelation using neighbourhood matrices. These matrices specify the relationship between the response values (in the case of Conditional Autoregressive CAR models) or residuals (in the case of Simultaneous Autoregressive SAR models) at each location *i *and those at neighbouring locations *j *[27]. SAR models can take three different forms depending on where the spatial autoregressive process is believed to occur [27]. The spatial error model (SAR_err_) assumes that the autoregressive process occurs only in the error term, and neither in response nor predictor variables. The lagged response model (SAR_lag_) assumes that the autoregressive process occurs in the response variable, and the lagged-mixed model (SAR_mix_) assumes that the spatial autocorrelation affects both response and predictor variables. Kissling and Carl [28] tested the performance of the three different SAR model types (SAR_err_, SAR_lag_, and SAR_mix_) and ordinary least squares (OLS) regression. They showed that SAR_err _models were the most reliable SAR models and that they performed well in all cases (independent of the kind of spatial correlation induced and whether models were selected by minRSA, R^2 ^or AIC), whereas OLS, SAR_lag _and SAR_mix _models showed weak type I error control and/or unpredictable biases in parameter estimates. Based on this conclusion we thus chose SAR_err _model to account for the spatial autocorrelation observed in the model residuals.

The neighbourhood relationship is formally expressed in a *n *× *n *matrix of spatial weights (W), with elements (w_ij_) representing a measure of the connection between locations *i *and *j*. Details on SAR models are provided elsewhere [see [27-29]]. Briefly, the usual ordinary least squares regression model Y = β X_i _+ ε is complemented by a term (λWμ), which represents the spatial structure (λW) in the spatially dependent error term (μ) [27]. The SAR_err _model thus takes the form Y = β X_i _+ λWμ + ε where λ is the spatial autoregression coefficient. We combined TSA with a spatial error simultaneous autoregressive (SAR_err_) model to account for spatial autocorrelation in the residuals [27,28]. This combination, hereafter referred to as trend SAR_err _model, leads to a model that takes the form

We estimated the best trend SAR_err _model using R software v.2.10.1 [30] and the spdep package [31]. We used a row standardized spatial weights matrix with a neighbourhood distance of 80 km. The neighbourhood distance of 80 km was chosen based on biological hypothesis as recommended by Dormann et al. [27]. The neighbourhood distance reflects the maximal distance at which the date of the first BT clinical case in a municipality influences the date of the first BT clinical case in surrounding municipalities. Based on the Commission Regulation No 1266/2007 the BT restricted zone, which represents the maximal distance at which contamination can occur from a BT infected farm, was defined as a 70-km radius around the infected farms. 70 km could thus be chosen as the neighbourhood distance to use in the spatial weights matrix. However, the minimal distance at which all municipalities of our dataset had at least one neighbour was 80 km. To avoid the problem of some areal entities having no neighbours [32] we chose a neighbourhood distance of 80 km, which is close to the 70-km radius of the restricted zone (see Additional file 1 for a description of the spatial weights matrix). Moran's I value, a measure of autocorrelation, and correlograms were calculated with the functions moran.test from the spdep package and correlog from the ncf package [33], respectively. Correlograms, which plot Moran's I values on the y-axis against geographic distance in the x-axis, allow the assessment of the spatial autocorrelation pattern with increasing distance. As recommended by Kissling and Carl [28], we computed two model selection statistics, the Akaike's Information Criterion (AIC) [34] and the minimum residual autocorrelation (minRSA). The latter was obtained by summing up the absolute Moran's I values in the first 80 distance classes of the correlogram [28]. The final model was chosen to minimize both AIC and minRSA based on backward selection. We considered that two nested models differing by less than 2 AIC points received identical support from the data. In such a situation, the model with less parameters was preferred [34].

To evaluate the selected model, a deviance-based pseudo-*R*^2 ^(in the following simply referred to as *R*^2^) was calculated as the squared Pearson correlation between predicted and observed values [28]. *R*^2 ^provides a measure of goodness of fit of the model. Observed Moran's I values, computed using the residuals from the trend SAR_err _model, provide a measure of the spatial autocorrelation in the model residuals [32,35].

The model predicted a date of the first clinical case occurrence in each contaminated municipality. These dates were then contoured using a contouring and splining function in the Spatial Analyst extension of ArcGIS software v.9.1 (ESRI Inc.). We used 30-day intervals for the contour lines so that each band would represent a month, which would facilitate comparison of disease spread between periods of time.

The velocities of the travelling waves of BT were calculated using the best-fit surface model. This model was used to derive partial differential equations ∂t/∂X and ∂t/∂Y [24]. Substituting the specific (X, Y) coordinates observed for a case into these equations allowed the X and Y geometric vectors contributing to the slope of the travelling wavefront (i.e., its magnitude) to be described [36]. For each municipality, we summed the X and Y geometric vectors to obtain a resultant slope vector with magnitude and direction. The inverse of the slope is the velocity or speed of diffusion of the epidemic at each municipality location in kilometers per day. The larger the velocity vector, the faster the speed of diffusion [24].

## Results

### Data description

**Figure 3:**
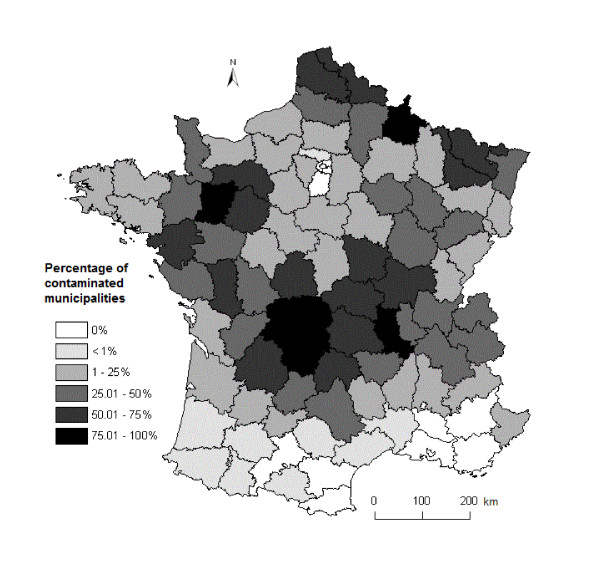
**Percentage of contaminated municipalities per department**. Contaminated municipalities are municipalities where at least one clinical case of BT due to the BTV-8 serotype was observed. A rate of 100% would indicate that a clinical case of BTV-8 was observed in all the municipalities of a department.

The date of the first clinical case of BTV-8 was considered as relevant for 10 994 municipalities in 82 departments. Descriptive statistics are provided in Table 1. The average number of municipalities per department was 415 (range: 102-894) with an average number of contaminated municipalities per department of 134 (1-533). The percentage of contaminated municipalities per department varied from 0.2 to 94% with a median value of 25.5% (Figure 3).

**Table 1:** Descriptive statistics of the data used for fitting a trend SARerr model of BT spread in France in 2007-2008, based on clinical cases of BTV-8.

variable	min	max	average	median
X	-743.9	168.2	-208.4	-197.8
Y	-868.2	26.3	-339.6	-328.5
*t*	270	715	491	556

X and Y are the geographic coordinates (in km) of the municipality centroids adjusted to the area of BT introduction, *t *is the number of days to the BT introduction in 2006 (excluding 90 days corresponding to the period from 1 January to 31 March), min and max, are the minimum and maximum values, respectively.

### The trend SAR_err _model

**Figure 4:**
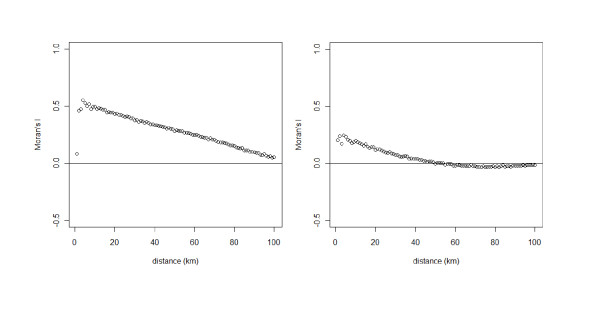
**Correlograms of the residuals from the TSA model (left) and the selected trend SAR_err _model (right)**. Correlogram plots Moran's I values on the y-axis against geographic distance in km in the x-axis. Moran's I has an expected value near zero for no spatial autocorrelation, with negative and positive values indicating negative and positive autocorrelation, respectively.

Based on backward selection from the full quadratic trend SAR_err _model we selected the model with the lowest AIC and minRSA (Table 2). All the trend SAR_err _models had lower AIC values and less spatial autocorrelation in the residuals (lower minRSA) than the simple TSA model, which did not account for spatial autocorrelation. Adding the spatial autocorrelation term to the TSA model also improved the model fit. Models m0 and m5 were similar in terms of AIC and minRSA (Table 2), but m5, which did not include a first order parameter for X, had the highest *R^2 ^*and the lowest Moran's I. Moreover, the values of the coefficients of m0 and m5 were similar (see Additional file 2), differing by less than 0.008, and the value of the estimate of the first order parameter for X in m0 was -0.0690, standard error = 0.0388, with a statistically non-significant *p*-value = 0.076. Based on these results we selected the model m5. This model provided an increase in *R^2 ^*from 0.85 to 0.96. Correlograms for the residuals of the TSA model and the selected trend SAR_err _model are showed in Figure 4. Although positive autocorrelation at small distances (less than 40 km) was still present in the residuals of the trend SAR_err _model, autocorrelation was greatly reduced in comparison to the TSA model. Moran's I values and minRSA decreased by 93% and 78%, respectively (Table 2).

**Table 2:** Summary characteristics from the trend SAR_err _model selection of BT spread in France in 2007-2008, based on clinical cases (*n *= 10 994 municipalities).

model	np	AIC	minRSA	*R^2^*	observed Moran's I
TSA	6	115 121	26.5	0.85	0.3894
m0	8	107 730	5.9	0.92	0.0285
m1	7	107 740	5.9	0.92	0.0298
m2	7	107 750	6.0	0.92	0.0319
m3	7	107 750	6.0	0.92	0.0309
m4	7	107 740	5.9	0.92	0.0299
m5	7	107 730	5.9	0.96	0.0282
m6	5	107 780	6.0	0.92	0.0345

Model selection was based on Akaike Information Criterion (AIC) and minimum residual spatial autocorrelation (minRSA). A measure of model fit and spatial autocorrelation in the model residuals are given as *R^2^; *and observed Moran's I, respectively. np is the number of parameters. m0 to m6 are the trend SAR_err _models, TSA model values are given for comparison. The selected trend SAR_err _model is m5.

TSA: *t *= β_0 _+ β_1_X + β_2_Y + β_3_X^2 ^+ β_4_XY + β_5_Y^2 ^+ ε

m0: *t *= β_0 _+ β_1_X + β_2_Y + β_3_X^2 ^+ β_4_XY + β_5_Y^2 ^+ λWμ + ε

m1: *t *= β_0 _+ β_1_X + β_2_Y + β_3_X^2 ^+ β_4_XY + λWμ + ε

m2: *t *= β_0 _+ β_1_X + β_2_Y + β_4_XY + β_5_Y^2 ^+ λWμ + ε

m3: *t *= β_0 _+ β_1_X + β_2_Y + β_3_X^2 ^+ β_5_Y^2 ^+ λWμ + ε

m4: *t *= β_0 _+ β_1_X + β_3_X^2 ^+ β_4_XY + β_5_Y^2 ^+ λWμ + ε

m5: *t *= β_0 _+ β_2_Y + β_3_X^2 ^+ β_4_XY + β_5_Y^2 ^+ λWμ + ε

m6: *t *= β_0 _+ β_1_X + β_2_Y + λWμ + ε.

**Figure 5:**
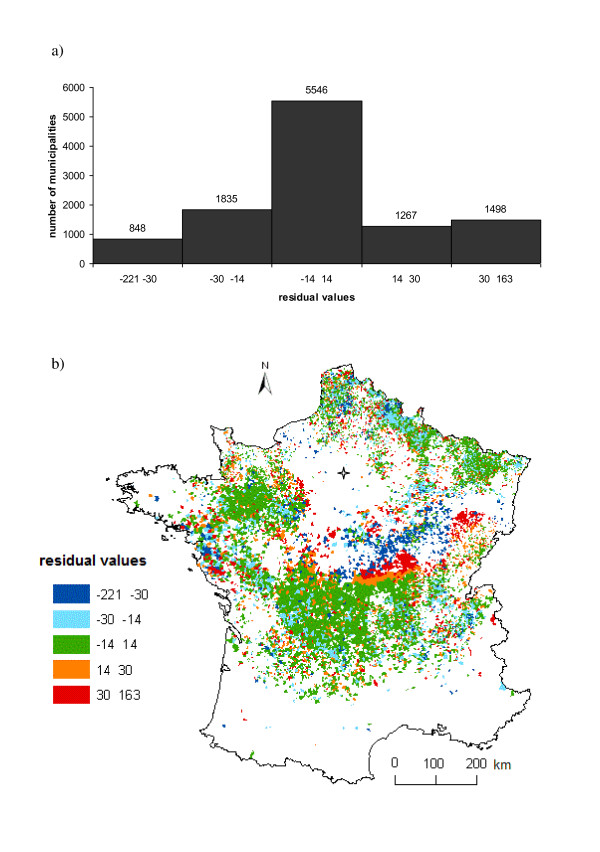
**Residual values of the final trend SAR_err _model**. a) Histogram of residual values. b) Map of residual values. Blank areas are municipalities that were not included in our dataset because of incomplete information or the absence of clinical cases. Paris is represented by a star. Areas of highest negative residual values (in dark blue) indicate a faster than predicted diffusion, while areas of highest positive residual (in red) indicate a slower than predicted diffusion.

The residuals of the final trend SAR_err _model had a mean non significantly different from zero (-2.1 e-12, 95% Confidence Interval (CI): -0.605-0.605) and a bell-shaped distribution (Figure 5a). To identify areas of earlier or later than predicted diffusion, the residuals of the final model were categorized into classes of residual values and plotted on the map of France (Figure 5b). An area of negative residual values (faster than predicted diffusion) in dark blue followed by an area of positive residual (slower than predicted diffusion) values in red are visually apparent in the centre of France. Overall, the difference between the predicted and observed date of the first clinical case occurrence was less than 30 days for 79% of the municipalities.

**Figure 6:**
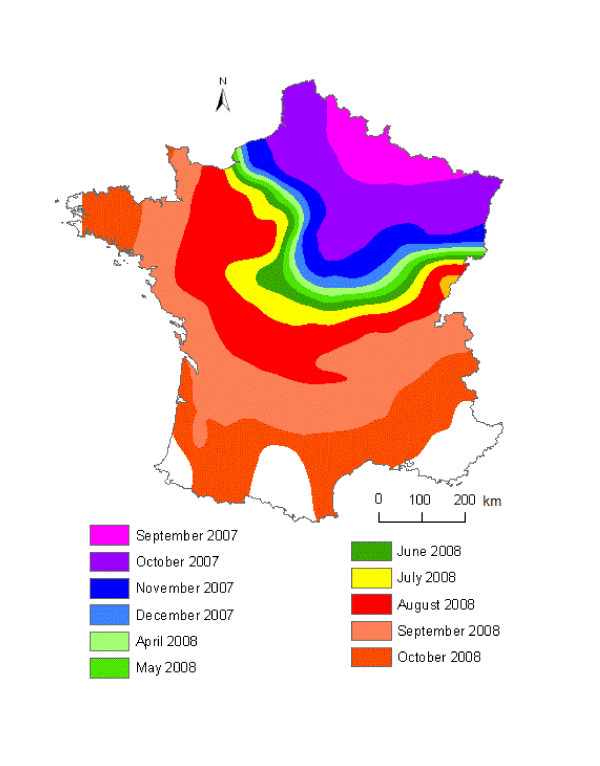
**Contour lines showing the trend SAR_err_-predicted date of first clinical case occurrence of BT**. A 30-day interval was used for the contour lines.

We used the final trend SAR_err _model to estimate the day of the first clinical case occurrence in each contaminated municipality, based on the geographic coordinates of the municipality centroids (Table 3). The contoured trend-surface map (Figure 6) of the predicted time of occurrence illustrates the general direction and movement of the diffusion of BT in France in 2007-2008, month by month. Contour lines that are far apart indicate that the disease spread rapidly through an area while lines close together indicate slow progression. The direction of diffusion is given by the front of the contour lines [24]. Overall, BT spread from north-eastern (introduction from Belgium and Germany) to south-western France. The disease spread slower during the less favourable months for *Culicoides *activity (Figure 6): November-December and April to July. The same pattern of disease spread was observed in 2007 and 2008 with the faster spread of BT occurring in August-October.

**Table 3:** Final trend SAR_err _model selected to explain the spread of BT across France in 2007-2008.

Predictor	Estimate	Standard Error	*p*
intercept	368.49	411.53	0.3706
Y	-0.1169	0.03717	0.0017
X^2^	3.0587 × 10^-4^	0.44053 × 10^-4^	< 0.0001
Y^2^	1.3306 × 10^-4^	0.46477 × 10^-4^	0.0042
XY	-3.2511 × 10^-4^	0.64416 × 10^-4^	< 0.0001

### Estimated velocity from the trend SAR_err _model

**Figure 7:**
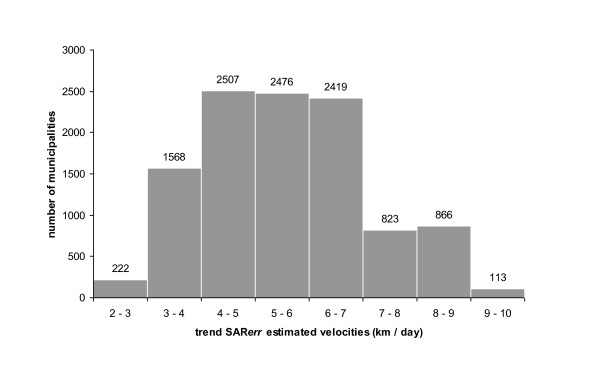
**Distribution of velocities of BT spread estimated from the trend SAR_err _model**. Velocities were estimated in the 10 994 French municipalities in which a clinical case were reported in 2007-2008.

The average estimated velocity across the country was 5.6 km/day. However, velocities varied according to areas and time period (Figure 6). Overall, estimated velocities of BT spread varied between 2.1 and 9.3 km/day (Figure 7). For 9192 municipalities (84%), trend SAR_err_-estimated velocities were lower than 7 km/day. 2% of the municipalities had an estimated velocity strictly lower than 3 km/day, which is the value commonly admitted of maximal active dispersal of *Culicoides *by flight [37].

## Discussion

We used a trend-SAR_err _model to assess the velocity of BTV-8 spread across France between 2007 and 2008 and compare the respective importance of local spread and long distance dissemination. The average estimated velocity of BT was 5.6 km/day, or 39.2 km/week. In line with our results, the velocity of BT spread in Sardinia during the Italian BTV-2 outbreak was about 30 km/week [38]. Our estimation is higher than the findings of Gerbier et al. [17], who found a 10-15 km/week rate of spread, during the early stage of the BTV-8 epidemics in The Netherlands, Germany, and Belgium. The difference could be due to the methods used in the two studies: whereas we corrected for spatial autocorrelated residuals, Gerbier et al. did not. Interestingly, we found a mean velocity of 11.9 km/week, a similar value than the findings of Gerbier et al. [17], when we did not corrected for spatial autocorrelation in the model residuals. The difference in the average estimated velocity of BT could also be related to the heterogeneity of landscape, host density, and altitude of our study area, as well as the broad range of period of time considered in our analysis. Indeed, we studied the speed of BT diffusion across France during two years whereas Gerbier et al. [17] focused only on the early stage of the epidemics, from 17 August to 15 September 2006, in a limited homogeneous rectangular area of 90 × 75 km.

The range of the estimated velocities suggests that BT transmission in France was primarily local: 84% of the contaminated municipalities had an estimated velocity lower than 7 km/day, and none above 10 km/day. Regarding long-range dissemination, *Culicoides *can be passively dispersed overseas over long distances (> 100 km) by prevailing wind [39-41]. Hendrickx et al. [39] developed wind models which suggested that long-distance spread over land of *Culicoides *by prevailing wind could have allowed the BT epidemics to cover large distances at a rate of 15 km/day. Here, with no velocity above 10 km/day, such long-range dispersal of infected *Culicoides *by wind was probably uncommon and had a negligible effect on BT spread. Considering that the flight range of *Culicoides *is short and most species disperse only at about 3 km from their breeding sites [37,42], active flight of *Culicoides *from farm to farm can not be the only factor explaining the local spread of BT. Indeed, the estimated velocity was less than 3 km/day for only 222 (2%) contaminated municipalities. Consequently, other factors must have facilitated the local diffusion of BT in France.

Several hypotheses can be proposed to explain these findings. The first hypothesis is that wind, besides facilitating the long-range dispersal of *Culicoides*, could have also increased the local dispersal of infected vectors [37,40,43]. Flight and orientation depends on the speed and direction of wind [43], but studies have shown that *Culicoides *prefer to shelter and cease almost all activity at wind speeds above 3 m/s or 10.8 km/h [37]. Interestingly, Bishop et al. [43] showed in Australia that high frequencies of wind speed above 8 km/h slowed the dispersion of *Culicoides brevitarsis*. Consequently, by stopping *Culicoides *activity, wind speeds above 3 m/s decrease local dispersal of infected vectors, whereas lower wind speeds could slightly increase the distance of local dispersal. Further analysis should investigate the wind patterns observed over the study area.

A second hypothesis is that dispersal distances may differ between *Culicoides *species. The maximal distance of active dispersal have only be estimated for North-American species: 3.2 km in 24 h for *Culicoides mississippiensis *[44], 4.8 km in 24 h for *Culicoides mohave *[45] and 5.6 km in 24 h for *Culicoides variipennis *[42]. Higher average distance of dispersal of the European species compared to the North-American ones could explain the velocities of BT spread estimated in our study. It will be important to determine whether dispersal capacities of European species are greater than the North-American species to support or invalidate this hypothesis.

Thirdly, local movements of infected animals may have facilitated BT diffusion around the contaminated farm units and have slightly increased the velocity of BT spread. Livestock are regularly moved within farms between pastures. However, little is known in literature on these movements. In France cattle farms have a mean surface area of 0.77 km^2^, which results in a mean radius of 0.5 km if we consider the farm as a circle. Brunschwig et al. [46] reported that in France dairy cows are moved in a 0.5 km radius around the farm, while beef cattle are regularly moved in a 5 km radius, and occasionally as far as 20 km from the farm. Regarding farm-to-farm cattle movements, in Great Britain Mitchell et al. reported a mean straight-line distance of 58 km with 43% of the movements occurring within less than 20 km [47], in Portugal 80% of the cattle trade movements were local (< 40 km) [48], and in Sweden 87% of the cattle movements were to farms within 100 km [49]. Overall, these studies showed that a large part of the cattle movements between farms occurs at distance less than 100 km. Restrictions on farm animals movements were implemented in France following the Directive 2000/75/EC, which defined the restricted zone for BT as a 150 km radius around the BT contaminated farm. In October 2007 the Commission Regulation No 1266/2007 reduced the restricted zone to a 70-km radius around the contaminated farms. While regulations on animal transport prevented any movements from the restricted zone to the non-restricted zones, movements between farms could occur within the restricted zone and may thus partly explain the range of observed velocities.

Finally, wild ungulates may also play a role in BT diffusion because i) high seroprevalence have been reported in cervid species, ii) similar patterns were reported in domestic herd and wild ungulates, and iii) experimental infections suggested that wild ungulates could be infectious to *Culicoides*. BTV antibodies have been reported in various wild cervid species in Europe [50-54]. In France, in 2008-2009 a mean BTV seroprevalence rate of 41% and 1% was found in red deer (*Cervus elaphus*) and roe deer (*Capreolus capreolus*), respectively [55]. In red deer seroprevalence rates varied from 8 to 70% according to the study areas. In Spain Ruiz-Fons et al. [50] found a seroprevalence rate of 22% in red deer and 5% in roe deer and Garcìa et al. [54] reported a seroprevalence rate of 66% in red deer. Furthermore, in Spain similar spatial and temporal BTV patterns were observed in red deer and livestock [50,54]. Moreover, the detection of BTV in skin samples of experimentally infected red deer suggests that they could be infectious to *Culicoides *[53]. Overall, red deer, in which BTV-infection does not induce a significant mortality [51], may serve as reservoir hosts. Movements of wild ungulate species could thus play a role in BT spread [54].

Although our data does not allow us to distinguish the effects of flight of *Culicoides *from the effects of short range movements of farm animals or wild ungulates (or any combination of the three) on BT spread, our results clearly show that BT diffusion in France was primarily local. Few measures were available in France to control the spread of the infection until the summer of 2008. While BTV-8 vaccines became available in the spring of 2008, and progressively were used in the second part of the year to protect livestock, regulations on animal transport probably were the most valuable measure in limiting the spread of BT through long-range dissemination [11].

TSA is one of numerous methods that can be used in the analysis of change over space. It mainly has been used in geological studies but we encourage researchers who are interested in a simple method to estimate the direction and speed of an infectious disease spread based solely on the geographical location and date of cases. However, investigators must be aware of the limitations of the method. A disadvantage of TSA is that predictions or extrapolations outside the area and time of study are not accurate and should not be made [21]. In our study, residual values of the TSA model showed high level of autocorrelation (Figure 4), but by combining the TSA model with a spatial error model, the observed Moran's I value decreased by 93%. The residuals of the trend SAR_err _model remained slightly positively autocorrelated at small distances (< 40 km). This may be due to i) the large dataset or ii) the omission of spatially patterned explanatory variables [27,32,56,57]. Indeed, Koenig stated [58]: "With large datasets, statistically significant values can be obtained even though the absolute degree of correlation is small; whether such low spatial autocorrelation is biologically significant or not must be considered on a case-by-case basis". Moreover, positive autocorrelation in the residuals at small distances can be observed when environmental factors associated to small-scale variations in the explained variable are omitted [56]. This paper shows that with simple epidemiological data (geographic coordinates and date of clinical cases), the TSA method enables to estimate and map the spread of a disease [24,36]. This first step can then be completed by modelling the effect of a wide range of environmental variables on the estimated velocities. Overall, both our large dataset (10 994 municipalities) and the use of geographic coordinates may explain the slightly autocorrelated residuals of the trend SAR_err _model. However, because i) TSA was specifically developed to fit spatial data, and ii) the remaining autocorrelation is small, we believe that it did not bias the estimated velocities. Unwin [22] noted that if the sample size is sufficiently large, and provided one avoids using a higher-order surface model, the TSA method is robust enough to allow some violation of the least-squares regression assumptions. Furthermore, the model was not use as a predictive tool to extrapolate the date of the first clinical case occurrence in other areas or at different periods of time. Overall, with a *R^2 ^*value of 0.96, and 79% of the municipalities for which the difference between the predicted and observed date of the first clinical case occurrence was less than 30 days, our model fitted the data well.

Another limitation of the TSA method is that interpretation at the edges of the map should be made with caution [24]. Residuals at the edges of the study area presented extreme positive and negative values (Figure 5b). The same pattern of extreme residual values also occurred around a geographical area in the centre of France where no or very few municipalities were included in our dataset, which induced an edge effect in the centre of the study area. The absence of BT infected municipalities in this area was due to the absence of, or to incomplete data on, clinical cases linked with low livestock densities. The trend SAR_err _model had problems handling such areas with no or few data and this led to areas with extreme residual values.

The pattern of autocorrelation in residual values can however be used to indicate heterogeneities in disease spread. Residuals should always be examined and lead to questions of why the disease occurred at a particular location earlier or later than predicted by the model [24]. In our case, most areas with extreme residual values in the centre of France (in red and dark blue in Figure 5b) correspond to the geographic areas where BT spread was very low immediately prior to and after the vector-free period (January to March 2008).

The 10 994 municipalities included in the study did not represent an exhaustive sample of municipalities with BT-infected animals. Indeed, some municipalities with clinical cases but incomplete data were excluded from the analysis, and we did not consider municipalities where they were no clinical cases but where seropositive farm animals were reported. However, while the case detection system based on clinical suspicion underestimated the real impact of the epidemic, it indicated a correct spatial trend [59].

In conclusion, TSA modelling procedure, combined with a spatial error model to limit the effect of autocorrelated residuals, is a powerful tool to provide information on the direction and speed of disease diffusion when the only data available are case date and location. Our study was a first step in describing the diffusion process for BT in France and showed that the spread of BT was primarily local. The next step in the spatial analytic process is to understand why BT spread the way it did. This will be achieved by using logistic regression to investigate the correlation between ecological factors, i.e., landscape pattern, farm animal density, meteorological conditions, and velocity of spread.

## Competing interests

The authors declare that they have no competing interests.

## Authors' contributions

MP participated in the design of the study, performed the statistical analysis and drafted the manuscript. HG, BD and DC participated in the design of the study and DA participated in the statistical analysis. CD conceived of the study, and participated in its design and coordination. All authors read, amended and approved the final manuscript.

## Supplementary Material

Additional file 1 **Description of the row standardized spatial weights matrix with the neighbourhood distance of 80 km**. Click here for file

Additional file 2 **Estimates, standard errors and *p*-values of m0 and m5 spatial models**. Click here for file
