# Supplementary material for: Estimating front-wave velocity of infectious diseases: a simple, efficient method applied to bluetongue
Source: Vet Res. 2011 Apr 20;42(1):60. doi: 10.1186/1297-9716-42-60 (PMC3090993; doi:10.1186/1297-9716-42-60)
Supplement: Additional file 2 — Estimates, standard errors and p-values of m0 and m5 spatial models. [file 1297-9716-42-60-S2.DOC]

Additional file 2:

Estimates, standard errors and *p*-values of the models m0 and m5.

| Predictor | Estimate | | Standard Error | | *p*-value | |
| --- | --- | --- | --- | --- | --- | --- |
|  | m5 | m0 | m5 | m0 | m5 | m0 |
| intercept | 368.49 | 383.31 | 411.53 | 414.65 | 0.3706 | 0.3553 |
| X |  | -0.0690 |  | 0.03888 |  | 0.0758 |
| Y | -0.1169 | -0.1248 | 0.03717 | 0.03744 | 0.0017 | 0.0009 |
| X² | 3.0587 × 10^-4^ | 2.5820 × 10^-4^ | 0.44053 × 10^-4^ | 0.51583 × 10^-4^ | < 0.0001 | < 0.0001 |
| Y² | 1.3306 × 10^-4^ | 1.4299 × 10^-4^ | 0.46477 × 10^-4^ | 0.46807 × 10^-4^ | 0.0042 | 0.0023 |
| XY | -3.2511 × 10^-4^ | -4.0407 × 10^-4^ | 0.64416 × 10^-4^ | 0.78273 × 10^-4^ | < 0.0001 | < 0.0001 |
